# Prevention of ventilator-associated pneumonia, mortality and all intensive care unit acquired infections by topically applied antimicrobial or antiseptic agents: a meta-analysis of randomized controlled trials in intensive care units

**DOI:** 10.1186/cc10285

**Published:** 2011-06-24

**Authors:** Claudia Pileggi, Aida Bianco, Domenico Flotta, Carmelo GA Nobile, Maria Pavia

**Affiliations:** 1 Department of Clinical and Experimental Medicine, Chair of Hygiene, Medical School, University of Catanzaro "Magna Græcia", via Tommaso Campanella, 88100 Catanzaro Italy; 2 Hospital Hygiene Unit, "Mater Domini" University Hospital, via Tommaso Campanella, 88100 Catanzaro Italy

## Abstract

**Introduction:**

Given the high morbidity and mortality attributable to ventilator-associated pneumonia (VAP) in intensive care unit (ICU) patients, prevention plays a key role in the management of patients undergoing mechanical ventilation. One of the candidate preventive interventions is the selective decontamination of the digestive or respiratory tract (SDRD) by topical antiseptic or antimicrobial agents. We performed a meta-analysis to investigate the effect of topical digestive or respiratory tract decontamination with antiseptics or antibiotics in the prevention of VAP, of mortality and of all ICU-acquired infections in mechanically ventilated ICU patients.

**Methods:**

A meta-analysis of randomised controlled trials was performed. The U.S. National Library of Medicine's MEDLINE database, Embase, and Cochrane Library computerized bibliographic databases, and reference lists of selected studies were used. Selection criteria for inclusion were: randomised controlled trials (RCTs); primary studies; examining the reduction of VAP and/or mortality and/or all ICU-acquired infections in ICU patients by prophylactic use of one or more of following topical treatments: 1) oropharyngeal decontamination using antiseptics or antibiotics, 2) gastrointestinal tract decontamination using antibiotics, 3) oropharyngeal plus gastrointestinal tract decontamination using antibiotics and 4) respiratory tract decontamination using antibiotics; reported enough data to estimate the odds ratio (OR) or risk ratio (RR) and their variance; English language; published through June 2010.

**Results:**

A total of 28 articles met all inclusion criteria and were included in the meta-analysis. The overall estimate of efficacy of topical SDRD in the prevention of VAP was 27% (95% CI of efficacy = 16% to 37%) for antiseptics and 36% (95% CI of efficacy = 18% to 50%) for antibiotics, whereas in none of the meta-analyses conducted on mortality was a significant effect found. The effect of topical SDRD in the prevention of all ICU-acquired infections was statistically significant (efficacy = 29%; 95% CI of efficacy = 14% to 41%) for antibiotics whereas the use of antiseptics did not show a significant beneficial effect.

**Conclusions:**

Topical SDRD using antiseptics or antimicrobial agents is effective in reducing the frequency of VAP in ICU. Unlike antiseptics, the use of topical antibiotics seems to be effective also in preventing all ICU-acquired infections, while the effectiveness on mortality of these two approaches needs to be investigated in further research.

## Introduction

Infections that develop during intensive care unit (ICU) stays represent a serious threat for critically ill patients since they affect about 30% of patients who are admitted to ICUs [1-6]. Ventilator associated pneumonia (VAP), defined as a parenchymal infection of the lung occurring in a patient who has been assisted by mechanical ventilation within the past 48 hours [7], is the most common infection acquired in the ICU [1]. VAP has a cumulative incidence of 10 to 25% and accounts for approximately 25% of all ICU infections and 50% of the antibiotics prescribed in ICU [8]. The impact of VAP is very high in terms of morbidity, complicating the course of 8 to 28% of the patients receiving mechanical ventilation [9,10], prolonged ICU stays by an average of 4.3 to 6.1 days [11-13] and attributable mortality rates that range from 5.8% to 27% [12-14]. Finally, VAP imposes excess costs to health care institutions, but a precise evaluation of such over-costs is difficult because it is dependent on different factors from one country to another, such as the health care system, organization of the hospital and costs of antibiotics [15].

Several studies strongly support the hypothesis that colonization of the aerodigestive tract is primarily involved in VAP and other ICU-acquired infections' pathogenesis, since micro-organisms move into the lower respiratory tract or, through the gut, into the blood or regional lymphatics [16-22]. For these reasons selective decontamination of the aerodigestive tract represents a main objective for infection prevention in ICU patients. Moreover, the endotracheal tube plays a major role in the occurrence of VAP by providing an abnormal continuum between the upper airway and the trachea and by establishing a subglottic reservoir of secretions containing large amounts of bacterial pathogens belonging to the oropharynx and the stomach [23]. Secretions are aspirated into the trachea and then disseminated into the lungs by the ventilator [24].

Given the high morbidity and mortality attributable to VAP, prevention plays a key role in the management of patients undergoing mechanical ventilation. Therefore, a number of studies have investigated the effect of the selective decontamination of the digestive or respiratory tract by topical antiseptic or antimicrobial agents in the reduction of VAP incidence; however, current guidelines from the Center for Disease Control (CDC) for preventing health-care-associated pneumonia, released in 2003 and never updated, classify these practices as an "unresolved issue" [16]. Moreover, this topic has also been analyzed in previous meta-analyses and systematic reviews [25-31]. These reviews included trials conducted until 2006 and demonstrated the effectiveness of topical antiseptics and antibiotics in reducing VAP. However, five studies [32-36] have been most recently published on the effectiveness of antiseptics in the prevention of VAP, and four of these [32-34,36] have come to opposite conclusions. Analogously, one additional trial [37] has been published on the role of topical antibiotics in the prevention of VAP that found no significant effectiveness of the treatment.

All the previously mentioned meta-analyses investigated, as a secondary outcome, the effects of topical antiseptics and antibiotics in the prevention of overall mortality, and they reported no significant effect of these interventions. Since then, five studies investigating the effect of topical antiseptics [32-36] and two of topical antibiotics [37,38] on the reduction of overall mortality have been published. Finally, several of the formerly quoted trials have investigated the role of topical antiseptics and antibiotics in the prevention of all ICU-acquired infections and have reported controversial results, whereas no meta-analyses have assessed this topic.

Therefore, we performed a meta-analysis with the following aims: our primary goal was to update meta-analyses on the effect of topical selective digestive (oropharyngeal alone or including gastrointestinal tract) or respiratory tract (subglottic area or trachea or aerosol in the respiratory loop) decontamination (SDRD) with antiseptics or antibiotics in the prevention of VAP in mechanical ventilated ICU patients; and our secondary goals were 1) to update meta-analyses on the effect of topical SDRD with antiseptics and antibiotics on mortality in mechanical ventilated ICU patients, and 2) to perform the first meta-analysis on the effect of topical SDRD with antiseptics or antibiotics on all ICU-acquired infections in mechanical ventilated ICU patients.

## Materials and methods

### Search strategy

A comprehensive systematic bibliographic search of medical literature published until June, 2010 was conducted to identify RCTs that assessed the effect of any type or combination of topical antibiotics or antiseptics on the prevention of pneumonia, all ICU-acquired infections and mortality in adults requiring mechanical ventilation in ICU.

The U.S. National Library of Medicine (MEDLINE), Embase and The Cochrane Library computerized bibliographic databases were used. In addition, we checked the references lists from all retrieved studies and meta-analyses or systematic reviews already published, to ensure that all studies could be identified. The following key words in different combinations were used: "aerosolized antibiotics", "airway colonization", "antibiotics", "antimicrobial prophylaxis", "antiseptic decontamination", "chlorhexidine", "critical care", "digestive decontamination", "healthcare-associated infections", "infection control", "intensive care units", "lower respiratory tract", "mechanical ventilation", "meta-analysis", "mortality", "nosocomial infection", "oropharyngeal decontamination", "pneumonia", "povidone-iodine", "prevention", "randomized controlled trials", "respiratory infection", "selective decontamination", "topical", "ventilator-associated pneumonia".

### Inclusion criteria

Articles that met the following criteria were included: (a) RCTs; (b) primary studies, not re-analyses or reviews; (c) examining the reduction of VAP and/or mortality and/or all ICU-acquired infections in mechanical ventilated ICU patients by prophylactic use of one or more of following topical treatments: 1) oropharyngeal decontamination using antiseptics or antibiotics, 2) gastrointestinal tract decontamination using antibiotics, 3) oropharyngeal plus gastrointestinal tract decontamination using antibiotics, 4) respiratory tract decontamination using antibiotics; (d) reported enough data to estimate the odds ratio (OR) or risk ratio (RR) and their variance; (e) English language; (f) published through June 2010. Trials that used systemic antibiotic prophylaxis were excluded.

### Assessment of study quality

Two of the authors independently reviewed the studies included in the meta-analysis to appraise the quality of the individual trial using criteria developed for Study Protocol and Data Analysis and Presentation by Chalmers *et al*. [39] and the method of Jadad *et al*. [40]. The Chalmers *et al*. scale assigns a weighting factor to each item according to whether it has been addressed completely (full score), partially (half score) or not at all (no score). If an item in the protocol was not applicable, the number of possible points was reduced. The final score of each paper was calculated as the total points scored divided by the total number of points thought applicable to that study, and two sub-scores regarding quality of Study Protocol and Data Analysis and Presentation were also calculated yielding a range from 0 to a full score of 1. The Jadad score, ranging from 0 to 5 points, was assigned to the included trials according to whether the investigators described the study as randomized and double-blind, reported the methods used to randomly assign patients and blind the intervention, and reported the number of withdrawals and dropouts and the reasons.

The readers discussed their evaluation and any disagreements were resolved through discussion and re-reading.

### Data extraction

The following items were collected from each clinical trial selected: a) study characteristics (authors, year of publication, design, length of follow-up); b) patients' characteristics (sample size in intervention and control groups, duration of mechanical ventilation, disease severity); c) VAP definitions and the incidence of VAP in treatment and control groups; d) all ICU-acquired infections' definitions and the incidence in treatment and control groups (we considered the number of patients who have developed at least one nosocomial infection including VAP); e) mortality in the ICU and/or in-hospital; f) type, concentration and mode of delivery of antimicrobial/antiseptics used, and control therapy.

Two independent reviewers extracted relevant trial characteristics and interobserver agreements were checked using the unweighted-kappa score [41]; differences between reviewers' data were resolved by discussion until a consensus was reached.

### Statistical analysis

The pooled effects estimates were used to combine the values from the single studies and were expressed as RR and the related 95% confidence intervals (CI). RR and CI were obtained using the Mantel-Haenszel fixed effects model [42], if the studies were homogeneous, and the DerSimonian and Laird random effect model [43] in cases with heterogeneity. Statistical heterogeneity was assessed using Cochran Q and I^2 ^measure; a I^2 ^value above 25% may be considered low heterogeneity, a value above 50% and 75% were predefined as moderate and high heterogeneity [44,45]. We preferred applying random effects models' results in case of I^2 ^equal or higher than 50%.

### Sensitivity analyses

The trials included in the meta-analysis differed considerably in several factors such as study design (method of randomization, blinding technique, modes of patients recruitment), clinical heterogeneity of patients (characteristics of participants, baseline disease severity), details of intervention (type and mode of administration of drugs, duration of treatment), and follow-up period, and subgroup analyses were used to explore eventual heterogeneity. We performed separate sensitivity analyses by grouping studies that had similar characteristics, such as patient population (medical or surgical or trauma or mixed critically ill patients); topical selective decontamination of the digestive tract or of the respiratory tract; associations of antimicrobial agents; antiseptic decontamination only with chlorhexidine; using placebo as comparator agent; double-blind studies. At last, we performed a meta-analysis to determine the potential impact of the quality of the studies on the results, by pooling only studies with Jadad scores greater than or equal to the median.

Finally, publication bias was explored by Egger's test [46,47] and Begg's rank correlation test [48]. All statistical analyses were performed using Stata software, version 10 (Stata Corporation 4905 Lakeway Drive, College Station, Texas 77845 USA) [49]. The reporting of the study's findings was in accordance with the PRISMA statement [50].

## Results

### Study characteristics

**Figure 1:**
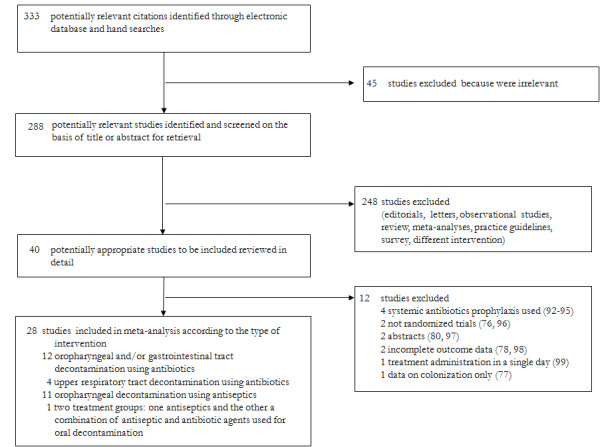
**Flow chart of the published trials evaluated for inclusion in the meta-analysis**.

A total of 333 publications were identified as potentially eligible for inclusion. Of these, 28 articles met all inclusion criteria and were included in the meta-analysis. A flow diagram providing the reasons for excluding the articles from the meta-analysis is reported in Figure 1. The agreement between the two researchers in the first comparison was 89%, with a kappa score of 0.88, and after discussion and detailed review of the articles was complete (k = 1).

Tables 1 and 2 summarize information on the patients and design of the included trials [32-38,51-71]. All trials assessed mortality as an outcome, 25 VAP [32-34,36,37,51-63,65-71] and 13 all ICU-acquired infections [37,38,51,52,54,56,58,60,64-68].

**Table 1:** Characteristics of included randomized controlled trials on topical decontamination by antiseptics

Authors	Country	Units of treatment T/C*	Interventions		Delivery mode	Daily amount and timing	Outcomes	Odds Ratio; *95% Confidence Interval*	Population
			**Treatment**	**Control**					
AJ De Riso *et al*. 1996 [51]	USA	173/180	0.12% CHX^† ^oral rinse + Standard oral care^‡^	Inert solution + Standard oral care^‡^	Oropharynx^§^	For 30 s 2 times/d	VAP^| ^In-hospital mortality All ICU^¶^-acquired infections	0.35; *0.1 to 1.26 *0.21; *0.05 to 0.94 *0.36; *0.16 to 0.77*	Cardiothoracic ICU^¶^
F Fourrier *et al*. 2000** [52]	France	30/30	0.2% CHX^† ^gel	Standard oral care^‡^	Dental and gingival surfaces	3 times/d	VAP^| ^Mortality All ICU^¶^-acquired infections	0.36; *0.13 to 1.01 *0.43; *0.12 to 1.5 *10; *2.59 to 42.21*	Multidisciplinary ICU^¶^
S Houston *et al*. 2002^†† ^[53]	USA	270/291	0.12% CHX^† ^oral rinse	Listerine^‡‡ ^oral rinse	Oropharynx^§,§§^	2 times/d For 10 days or until extubation	VAP^| ^Mortality	0.48; *0.15 to 1.54 *2.16; *0.54 to 8.53*	Cardiothoracic ICU^¶^
F Fourrier *et al*. 2005 [54]	France	114/114	0.2% CHX^† ^gel	Placebo gel	Dental and gingival surfaces	3 times/d until 28 days	VAP^| ^ICU^¶ ^mortality All ICU**-acquired infections	1.08; *0.52 to 2.27 *1.29; *0.81 to 2.06 *1.06; *0.51 to 2.21*	Multidisciplinary ICU^¶ ^
P Seguin *et al*. 2006^†† ^[55]	France	36/62	10% povidone-iodine oral rinse+ aspiration of oropharyngeal secretions	31 saline group^|| ^31 control group^¶¶^	Oropharynx^§ ^and nasopharynx	Every 4 hours	VAP^| ^ICU^¶ ^mortality	0.21; *0.07 to 0.64 *0.65; *0.28 to 1.5*	Surgical ICU^¶ ^
P Segers *et al*. 2006 [56]	Netherland	485/469	0.12% CHX^† ^oral rinse and nasal gel	Placebo oral rinse and nasal gel	Oropharynx^§ ^and nasal cavities	Oral rinse for 30 s 4 times/d; nasal gel 4 times/d	VAP^| ^In-hospital mortality All ICU^¶^-acquired infections	0.59; *0.42 to 0.83 *1.1; *0.39 to 3.15 *0.58*; 0.44 to 0.78*	Cardiothoracic ICU^¶^
M Koeman *et al*. 2006 [57]	Netherland	127/130	2% CHX^† ^in vaseline	Vaseline	Buccal cavity	4 times/d	VAP^| ^ICU^¶ ^mortality	0.58; *0.31 to 1.09 *1.12; *0.72 to 1.17*	Multidisciplinary ICU^¶^
H Tantipong *et al*. 2008^†† ^[32]	Thailand	102/105	Oral care^††† ^with 2% CHX^† ^solution	Oral care***with normal saline solution	Oropharynx^§^	4 times/d	VAP^| ^Mortality	*0.58; 0.27 to 1.22 *1*;0.75 to 1.34*	Multidisciplinary ICU^¶ ^General medical ward
F Bellissimo-Rodrigues *et al*. 2009 [33]	Brazil	98/96	0.12% CHX^† ^oral rinse	Placebo oral rinse	Buccal cavity	3 times/d until ICU discharge	VAP^| ^ICU^¶ ^mortality	0.91; *0.39 to 2.06 *1.06; *0.56 to 1.99*	Multidisciplinary ICU^¶^
TS Panchabhai *et al*. 2009^†† ^[34]	India	88/83	Cleansing^†††^with 0.2% CHX^† ^+ normal saline solution	Cleansing^†††^with 0.01% PP^‡‡‡^+ normal saline solution	Oropharynx^§ ^and hypopharynx	2 times/d until ICU discharge or death	VAP^| ^In-hospital mortality	0.88; *0.45 to 1.71 *1.18; *0.96 to 1.46*	Multidisciplinary ICU^¶^
CL Munro *et al*. 2009^†† ^[35]	USA	44/51	0.12% CHX^† ^oral swab	Oral care (not specified)	Buccal cavity	2 times/d	In-hospital mortality	*1.96; 0.67 to 5.87*	Multidisciplinary ICU^¶^
F Scannapieco *et al*. 2009 [36]	USA	116^§§§^/59	Standard oral care^|||^+ 0.12% CHX^† ^oral rinse	Standard oral care^|||^+ Placebo oral rinse	Buccal cavity	2 times/d	VAP^| ^ICU^¶ ^mortality^¶¶¶ ^	0.54; *0.23 to 1.25 *1.01; *0.37 to 2.97*	Trauma ICU^¶^

* Treatment/control

^†^chlorhexidine gluconate

^‡ ^mouth rinsing with bicarbonate isotonic serum followed by oropharyngeal aspiration four times a day

^§ ^buccal, pharyngeal, gingival, tongue and tooth surfaces

^| ^ventilator associated pneumonia

**^¶ ^**intensive care units

**single blind randomized study

**^†† ^**not blind randomized study

^‡‡ ^phenolic mixture

^§§^patients received 15 ml of the experimental or the control drug preoperatively

^||^saline solution oral rinse followed by oropharyngeal aspiration

^¶¶^regimen without any instillation but with aspiration of oropharyngeal secretions only

*** patients received teeth brushing, oral secretions suctioning and the oropharyngeal mucosa rubbing with solution

^††† ^oropharyngeal secretion suction and swab of the oral cavity, teeth, palate, buccal spaces, posterior pharyngeal wall and hypopharynx with normal saline solution followed by the same procedure with one of the two study solutions

^‡‡‡^potassium permanganate

^§§§^two arms in treatment group: 58 patient received once daily chx and once daily placebo; 58 patients received twice daily chx

^|||^suction toothbrush twice a day and swabbing every four hours

^¶¶¶^treatment group: 97; control group: 49

**Table 2:** Characteristics of included randomized controlled trials on topical decontamination by antibiotics

Authors	Country	Units of treatment T/C*	Interventions		Delivery mode	Daily amount and timing	Outcomes	Odds Ratio; *95% Confidence Interval*	Population
			**Treatment**	**Control**					
J Klastersky *et al*. 1974 [58]	Belgium	43/42	Gentamicin (S)	Normal saline (S)	Trachea	3 times/d	VAP^† ^Mortality All ICU^§^-acquired infections	0.38; *0.17 to 0.86 *1.36; *0.89 to 2.07 *0.43; *0.16 to 1.14*	Neurosurgical ICU^§^
K Unertl *et al*.^| ^1987 [59]	Germany	19/20	Polymyxin B+ Gentamicin (S) Amphotericin B (Su)	No antimicrobial prophylaxis	S applied orally, nasally and enterally; Su in the oropharynx^¶ ^(only T group)	4 times/d	VAP^† ^Mortality	0.12; *0.02 to 0.84 *0.91; *0.43 to 1.92*	Multidisciplinary ICU^§ ^
C Brun Buisson** *et al*. 1989 [60]	France	36/50	Disinfection ^††^+ Polymyxin E+ Neomycin+ Nalidixic acid (S)	Disinfection^††^	Disinfection^†† ^of oropharynx^¶^; S applied orally and enterally	Disinfection^†† ^3 times/d; S 4 times/d	VAP^† ^ICU^§ ^mortality All ICU^§^-acquired infections	0.69; *0.19 to 2.59 *0.94*; 0.51 to 1.73 *0.97; *0.35 to 2.63*	Medical ICU^§^
JM Rodriguez-Roldan *et al*. 1990 [61)	Spain	13/15	Disinfection^‡‡^+ Polymyxin E+ Tobramycin or Netilmicin+ Amphotericin B (P)	Disinfection^‡‡^+ Inert coloring substance (P)	Oropharynx^¶^	4 times/d	VAP^† ^In-hospital mortality	0.05; *0.0 to 0.77 *0.92; *0.31 to 2.73*	Multidisciplinary ICU^§ ^
J Pugin *et al*. 1991 [62]	Switzerland	25/27	Polymyxin B+ Neomycin+ Vancomycin (S)	Dextrose 5% (S)	Unconscious patients: instilled into retropharynx. Conscious patients: keep the solution in buccal cavity for 1 minute and then to shallow it	Every 24 h	VAP^† ^In-hospital mortality	0.21; *0.08 to 0.52 *1.08; *0.44 to 2.64*	Surgical ICU^§^
H Gastinne *et al*. 1992 [63]	France	220/225	Colistin+ Tobramycin+ Amphotericin B (S,G)	Nonabsorbable calcium salt (S, G)	G in oropharynx^¶^; S enterally	4 times/d	VAP^† ^In-hospital mortality	1.3; *0.8 to 2.1 *1.08; *0.89 to 1.3*	Medical ICU^§^
FB Cerra *et al*. 1992 [64]	USA	25/21	Norfloxacin (Su) + Nystatin (Su)	Cherry syrup (Su)	Enterally	Norfloxacin × 3 Nystatin ×4 limited to 15 d	ICU^§ ^mortality All ICU^§^-acquired infections	1.08; *0.64 to 1.84 *0.67; *0.41 to 1.1*	Surgical ICU^§^
AM Korinek *et al*. 1993 [65]	France	63/60	Polymyxin E+ Tobramycin+ Amphotericin B (S) and P containing same antibiotics plus Vancomycin	Sterile water (S) Carboxymethylcellulose (P)	P in oropharynx^¶ ^S administered enterally	4 times/d limited to 15d	VAP^† ^ICU^§ ^mortality In-hospital mortality All ICU^§^-acquired infections	0.57; *0.34 to 0.97 *0.57; *0.22 to 1.48 *1.09; *0.59 to 2.01 *0.56; *0.42 to 0.76*	Neurosurgical ICU^§^
J Wiener *et al*. 1995 [66]	USA	30/31	Polymyxin E+ Gentamicin+ Nystatin (S, P)	Inert S and P	P in oropharynx^¶ ^S administered enterally	4 times/d	VAP^† ^ICU^§ ^mortality All ICU^§^-acquired infections	1.03; *0.45 to 2.4 *0.78; *0.45 to 1.34 *0.82; *0.27 to 2.53*	Multidisciplinary ICU^§ ^
B Quinio *et al*. 1996 [67]	France	76/72	Polymyxin E+ Gentamicin+ Amphotericin B (Su,P)^§§^	Carboxymethylcell0ulose (Su, P)^§§^	G in oropharynx^¶ ^S administered enterally	4 times/d	VAP^† ^ICU^§^mortality All ICU^§^-acquired infections	0.49; *0.31 to 0.76 *1.12; *0.75 to 1.67 *0.6; *0.49 to 0.75*	Multiple trauma patients admitted in ICU^§^
DCJJ Bergmans *et al*. 2001 [68]	Netherland	87/139	Polymyxin E+ Gentamicin+ Vancomycin (O)	O without antibiotics^||^	Buccal cavity	Every 6 h limited to 21d	VAP^† ^ICU^§ ^mortality In-hospital mortality All ICU^§^-acquired infections	0.37; *0.19 to 0.74 *0.65; *0.35 to 1.21 *0.71; *0.39 to 1.29 *0.61;*0.34 to 1.1*	Multidisciplinary ICU^§ ^
GC Wood *et al*. 2002 [69]	USA	20/20	Ceftazidime (A)	Normal saline (A)	Nebulizer connected to the inspiratory loop	Every 12 hours for ≥ 7d	VAP^† ^Mortality	0.47; *0.23 to 0.98 *0.41; *0.06 to 2.41*	Trauma ICU^§^
I Pneumatikos *et al*. 2002**[70]	Greece	31/30	Polymyxin E+ Tobramycin+ Amphotericin B (S)	Placebo S	Subglottic area	Continuous infusion	VAP^† ^Mortality	0.37; *0.17 to 0.81 *0.63; *0.14 to 2.7*	Multiple trauma patients admitted in ICU^§^
M Koeman *et al*. 2006 [57]	Netherland	128/130	CHX^¶¶ ^+ Colistin in vaseline	Vaseline	Buccal cavity	4 times/d	VAP† ICU^§ ^mortality	0.82; *0.41 to 1.63 *1.02; *0.66 to 1.59*	Multidisciplinary ICU^§ ^
M Kollef *et al*. 2006 [71]	Multinational study***	362/347	Iseganan (S)	Placebo S	Oropharynx^¶^	For 2 min 6 times/d limited to 14d	VAP^† ^ICU^§ ^mortality at 14d	0.86; *0.68 to 1.09 *1.28; *0.87 to 1.88*	Multidisciplinary ICU^§ ^
JA Claridge *et al*. 2007 [37]	USA	53/52	Ceftazidime (A)	Normal saline(A)	Nebulizer connected to the inspiratory loop	Every 12 hours for ≥ 7d	VAP^† ^Mortality All ICU^§ ^-acquired infections^†††^	0.98; *0.67 to 1.43 *1.08; *0.63 to 1.85 *1.71; *0.67 to 4.48*	Trauma ICU^§^
AM de Smet *et al*. 2009** [38]	Netherland	1904/1990	Polymyxin E+ Amphotericin B+ Tobramycin (P)	Standard oral care^‡‡‡^	Buccal cavity (only T group)	4 times/d	In-hospital mortality ICU^§ ^mortality Mortality at day 28 All ICU^§^-acquired infections^§§§^	0.95; *0.83 to 1.09 *0.98; *0.84 to 1.15 *0.96; *0.74 to 0.99 *0.68; *0.53 to 0.86*	Multidisciplinary ICU^§ ^

A, aerosol; g, gel; o, orabase; p, paste; s, solution; su, suspension

* treatment/control, ^† ^ventilator associated pneumonia, ^§ ^intensive care units, ^| ^randomized study blinded for radiologic diagnosis, ^¶ ^buccal, pharyngeal, gingival, tongue and tooth surfaces, ** not blind randomized study, ^†† ^disinfection with a povidone-iodine solution, ^‡‡ ^disinfection with a 0.1% chlorhexidine solution, ^§§ ^both groups received nasal and oropharyngeal toilet with povidone-iodine before each treatment or placebo application, ^|| ^two separate control groups: control a (78) was studied in the presence of patients receiving topical antimicrobial prophylaxis; control b (61) was studied in ICU where no topical antimicrobial prophylaxis was used

^¶¶ ^chlorhexidine gluconate, *** France, Spain, Switzerland, Netherland, UK, USA, ^††† ^multi-drug-resistant infections, ^‡‡‡ ^mouth rinsing with water 4 times a day and tooth brushing twice daily, ^§§§ ^patients with at least one episode of bacteremia or candidemia acquired in ICU.

### Data quality

The mean quality scores of the individual studies using the Chalmers *et al*. scale was extremely variable, ranging from 0.19 to 0.9 (mean = 0.64), for the Protocol from 0.25 to 0.97 (mean = 0.61) and for the Data Analysis and Presentation from 0.13 to 0.88 (mean = 0.58). It should be noted that none of the studies had a full score, both for Protocol and Data Analysis and Presentation. Almost all trials received full credit for description of daily amount and timing of therapeutic regimens (93%), presentation of test statistic and *P-*value (93%) and number of patients who withdrew and the reasons why (89%), most trials reported description of relevant variables in experimental and control group (86%), criteria for patient selection (82%), methods for assuring masking of randomization and to evaluate success of masking (75%) and again start and stop dates (71%) and analysis of comparability of the study groups (61%). A few studies reported criteria for stopping the trial (39%), CI (36%) and only 18% and 21% of the studies discussed beta error and side effects of treatment, respectively (Table 3). With regard to the Jadad *et al*. criteria, the mean score was 3.39 (median 4), most trials addressed adequately the problems of withdrawals or dropouts after randomization (74%) and were classified as double-blinded (63%). Only eight trials [33,36,56,57,64,66,68,71] had a full score on the Jadad *et al*. scale. It should be noted that there has been improvement in study design and reporting, since findings published more recently tended to receive a higher quality rating.

**Table 3:** Distribution of studies by quality scoring values according to the Chalmers *et al*. method

Quality items	Adequate*	
**Research protocol**	**N**.	**%**
Description of inclusion and rejection criteria for patient selection (28)	23	82
Number and description of patients eligible not accepted (28)	16	57
Daily amount and timing of therapeutic regimen (28)	26	93
Physical appearance of placebo/control similar to the treatment (21)	16	76
Taste of placebo/control similar to the treatment (21)	10	48
Description and appropriate use of methods for assuring masking of randomization (28)	21	75
Patients masked treatment (27)	18	67
Observers masked to treatment (27)	17	63
Observers masked to results (28)	6	21
Prior estimate of sample size and power calculation (28)	15	54
Definition of criteria for stopping the trial (28)	11	39
Test of validity of randomization through description of relevant demographic and prognostic variables in experimental and control group (28)	24	86
Methods used to evaluate success of masking (20)	15	75
Methods used to ascertain compliance to treatment (0)	-	-
Laboratory tests to evaluate absorption or pharmacological effect of the treatment (16)	2	13
More than one observer evaluating subjective endpoints (23)	2	19
**Data analysis and presentation**		
Start and stop dates (28)	20	71
Analysis of results of randomization through baseline comparability of the study groups (28)	17	61
Presentation of test statistics and *P-*value (28)	26	93
Discussion of ß error in negative trials (17)	3	18
Calculation of estimate of variance and/or confidence limits of trials endpoints (28)	10	36
Regression/correlation analysis (25)	12	48
Overall assessment of quality of statistical analysis (28)	1	4
Number of patients who withdrew and the reasons why (28)	25	89
Ways withdrawals were handled (23)	1	4
Side effects reported and analyzed (28)	6	21
Analysis of subgroups not specified at the beginning of the study (retrospective analysis) (28)	2	7

Number of studies for which item was applicable in parenthesis

*Completely addressed the issue

### Meta-analysis

Results of the meta-analyses that explored the effects of topical use of antiseptics and antibiotics on the prevention of VAP, mortality and all ICU-acquired infections are shown in Table 4.

**Table 4:** Meta-analysis results of effectiveness of topical decontamination in reducing VAP, mortality and all ICU-acquired infections

	Antiseptics					Antibiotics				
**VAP*^† ^*Prevention**	**No. Studies**	**No. Patients**	**Overall Risk Ratio (Efficacy**, **%)**	**95% Confidence Interval (Efficacy interval,%)**	**Heterogeneity Test (*Q; P*; I^2^,%)**	**No. Studies**	**No. Patients**	**Overall Risk Ratio (Efficacy,%)**	**95% Confidence Interval (Efficacy interval, %)**	**Heterogeneity Test (*Q; P*; I^2^,%)**
**All studies**	11	**3,258**	0.73 (27)	0.63 to 0.84 (16 to 37)	*13.78; *0.18; 27.4	15	**2,463**	0.64 (36)	0.5 to 0.82 (18 to 50)	*47.09; <*0.001; 70.3
**High quality**	6	**2,161**	0.77 (23)	0.66 to 0.9 (10 to 34)	*4.62; *0.46; 0	11	**2,249**	0.69 (31)	0.54 to 0.89 (11 to 46)	*34.66; <*0.001; 71.2
**Low quality**	5	**1,097**	0.6 (40)	0.44 to 0.82 (18 to 56)	*8.99; *0.06; 55.5	4	**214**	0.35 (65)	0.14 to 0.86 (14 to 86)	*5.64; *0.13; 46.8
Only specialty surgery ICU*	4	**1,966**	0.52 (48)	0.38 to 0.71 (29 to 62)	*3.55; *0.31; 15.5	3	**260**	0.4 (60)	0.2 to 0.8 (20 to 80)	*5.64; *0.06; 64.6
**Only trauma patients**	NA^‡^	**-**	-	-	-	4	**354**	0.6 (40)	0.38 to 0.93 (7 to 62)	*8.2; *0.04; 63.4
Mixed ICU*	6	**1,117**	0.82 (18)	0.68 to 1.00 (0 to 32)	*5.77; *0.33; 13.3	6	**1,318**	0.7 (30)	0.46 to 1.05 (-5 to 54)	*13.41; *0.02; 62.7
**Only double blinded studies**	Same high quality meta-analysis					12	**2,277**	0.67 (33)	0.52 to 0.87 (13 to 48)	*39.53; <*0.001; 72.2
**Not double blinded studies**	Same low quality meta-analysis					3	**186**	0.44 (56)	0.21 to 0.92 (8 to 79)	*2.83; *0.24; 29.3
**Using same antibiotics combination:**										
**Cyclic peptide + aminoglycoside + polyene antifungal drug**	-	**-**	-	-	-	6	**782**	0.60 (40)	0.35 to 1.04 (-4 to 65)	*22.21; <*0.001; 77.5
**Vancomycin + other antimicrobial agents **	-	**-**	-	-	-	3	**401**	0.43 (57)	0.24 to 0.78 (22 to 76)	*5.3; *0.07; 62.3
**Digestive tract decontamination with antibiotics**	-	**-**	-	-	-	11	**2,172**	0.67 (33)	0.5 to 0.9 (10 to 50)	*35.99; <*0.001; 72.2
**Respiratory tract decontamination with antibiotics**	-	**-**	-	-	-	4	**291**	0.54 (46)	0.3 to 0.97 (3 to 97)	*9.67; *0.02; 69
**Mortality**										
**All studies**	12	**3,224**	1.1 (-10)	0.98 to 1.24 (-24 to 2)	*9.89; *0.54; 0	17	**6,403**	1.02 (-2)	0.93 to 1.13 (-13 to 7)	*9.82; *0.88; 0
**High quality**	6	**2,132**	1.09 (-9)	0.9 to 1.32 (-32 to 10)	*3.59; *0.61; 0	12	**2,295**	1.06 (-6)	0.94 to 1.2 (-20 to 6)	*8.55;*0.66;0
**Low quality**	6	**1,192**	1.11 (-11)	0.96 to 1.29 (-29 to 4)	*6.27; *0.28; 20.3	5	**4,108**	0.97 (3)	0.84 to 1.12 (-12 to 16)	*0.38; *0.98; 0
Only ICU* mortality	8	**1,751**	1.08 (-8)	0.92 to 1.26 (-26 to 8)	*4.9; *0.67; 0	14	**5,878**	1.01 (-1)	0.9 to 1.12 (-12 to 10)	*9.39; *0.74; 0
**Only in-hospital mortality**	4	**1,573**	0.95 (5)	0.95 to 1.37 (-37 to 5)	*4.74; *0.19; 36.7	6	**4,768**	0.98 (2)	0.88 to 1.09 (-9 to 12)	*2.43; *0.79; 0
**Only double blinded studies**	Same high quality meta-analysis					13	**2,323**	1.06 (-6)	0.94 to 1.2 (-20 to 6)	*8.59; *0.74; 0
**Not double blinded studies**	Same low quality meta-analysis					4	**4,080**	0.97 (3)	0.84 to 1.12 (-12 to 16)	*0.37; *0.95; 0
**Using same antibiotics combination:**										
**Cyclic peptide + aminoglycoside + polyene antifungal drug**	-		-	-	-	7	**4,676**	0.99 (1)	0.89 to 1.09 (-9 to 11)	*2.62; *0.85; 0
**Vancomycin + other antimicrobial agents **	-	**-**	-	-	-	3	**401**	0.89 (11)	0.59 to 1.35 (-35 to 41)	*1.06; *0.59; 0
**Digestive tract decontamination with antibiotics**	-	**-**	-	-	-	13	**6,112**	1.01 (-1)	0.92 to 1.12 (-12 to 8)	*6.72; *0.87; 0
**Respiratory tract decontamination with antibiotics**	-	**-**	-	-	-	4	**291**	1.17 (-17)	0.85 to 1.61 (-61 to 15)	*2.36; *0.5; 0
All ICU*-Acquired infections										
**All studies**	4	**1,595**	1.02 (-2)	0.41 to 2.51 (-151 to 59)	*20.14; <*0.001; 85.1	9	**4,774**	0.71 (29)	0.59 to 0.86 (14 to 41)	*18.62; *0.02; 57
**High quality**	3	**1,535**	0.59 (41)	0.47 to 0.76 (0.24 to 53)	*3.9; *0.14; 48.7	7	**794**	0.64 (36)	0.56 to 0.73 (27 to 44)	*7.57; *0.27; 20.7
**Low quality**	NA^‡^	**-**	-	-	-	2	**3,980**	0.89 (11)	0.52 to 1.52 (-52 to 48)	*6.57; *0.01; 84.8
Only specialty surgery ICU*	2	**1,307**	0.55 (45)	0.43 to 0.72 (28 to 57)	*1.19; *0.28; 16	4	**340**	0.71 (29)	0.45 to 1.11 (-11 to 55)	*12.15; *0.007; 75.3
Mixed ICU*	2	**288**	3.02 (-202)	0.34 to 27.12 (-2.61 to 66)	*8.2; *0.004; 87.8	3	**4,181**	0.7 (30)	0.59 to 0.85 (15 to 41)	*0.65; *0.72; 0
**Using same antibiotics combination:**										
**Cyclic peptide + aminoglycoside + polyene + antifungal drug**	-	**-**	-	-	-	3	**4,103**	0.66 (34)	0.57 to 0.76 (24 to 43)	*1.91; *0.38; 0
**Vancomycin + other antimicrobial agents **	-	**-**	-	-	-	2	**349**	0.53 (43)	0.44 to 0.74 (26 to 56)	*0.07; *0.79; 0
**Digestive tract decontamination with antibiotics**	-	**-**	-	-	-	7	**4,584**	0.7 (30)	0.58 to 0.84 (16 to 42)	*14.11; *0.03; 57.5
**Respiratory tract decontamination with antibiotics**	-	**-**	-	-	-	2	**190**	0.87 (13)	0.22 to 3.35 (-235 to 78)	*3.95; *0.05; 74.7

* Intensive care units, ^† ^ventilator associated pneumonia, ^‡^not applicable because only one study belongs to this group.

#### VAP

**Figure 2:**
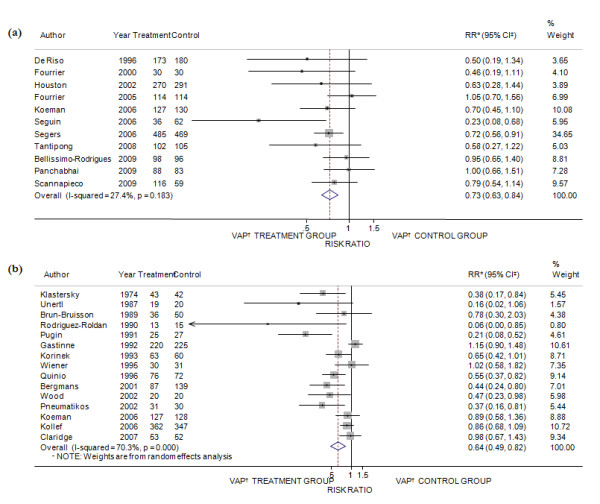
**Meta-analysis of the effectiveness of topical SDRD in the prevention of VAP in ICU**. (**a**) Decontamination by antiseptics. (**b**) Decontamination by antibiotics. * Risk ratio; ^‡ ^confidence interval; ^† ^ventilator associated pneumonia.

The incidence reduction of VAP in ICU patients by topical SDRD was the main outcome measured. Results from 11 trials [32-34,36,51-57] were available for the analysis of the effects of topical digestive decontamination with antiseptics (Figure 2). The overall estimate of efficacy of antiseptics in the fixed effects model was 27% (95% CI of efficacy = 16% to 37%) and the I^2 ^statistic test of homogeneity found a low heterogeneity across the various studies (Q = 13.78 *P *= 0.18; I^2 ^= 27.4%). Meta-analysis of the 15 trials that tested the effect of topical SDRD by antibiotics [37,57-63,65-71] (Figure 2) found a similar (36%) statistically significant reduction in VAP rates (95% CI of efficacy = 18% to 50%) but the test of homogeneity showed a moderate degree of statistically significant heterogeneity (Q = 47.09 *P *< 0.001; I^2 ^= 70.3%).

The results of the meta-analyses after limiting the analysis to high or low quality studies were not substantially changed, although the efficacy was higher in the low quality studies that used antiseptics and antibiotics as compared to that found in the high quality trials both with antiseptics and antibiotics agents.

Sensitivity analyses were conducted to determine whether the efficacy of chlorhexidine was correlated to dose (0.12% vs 0.20% or 2% preparations; twice a day application vs three or four applications a day). The results showed a 31% significant incidence reduction of VAP (95%CI of efficacy = 3% to 51%; Q = 0.91 *P *= 0.63; I^2 ^= 0%) even using the lowest concentration of chlorhexidine (twice a day application of 0.12% chlorhexidine gluconate) (data not shown).

Pooled analysis after restriction to specific settings showed that, both for antiseptics and antibiotics, the highest efficacy was found in specialty surgical ICUs and the lowest in mixed ICUs.

The separate meta-analyses involving digestive tract decontamination by antibiotics showed lower efficacy as compared to respiratory tract decontamination by antibiotics.

Finally, we tested the effectiveness of similar combinations of topical antibiotics on VAP prevention and only in the trials that used vancomycin combined with other antimicrobial agents, a significant VAP reduction was found. Overall, low heterogeneity was revealed in almost all sensitivity analyses involving antiseptics, whereas a significant heterogeneity remained in the results among trials on antibiotic prophylaxis of VAP.

#### Mortality

In none of the meta-analyses conducted on mortality was a significant effect found.

#### All ICU-acquired infections

**Figure 3:**
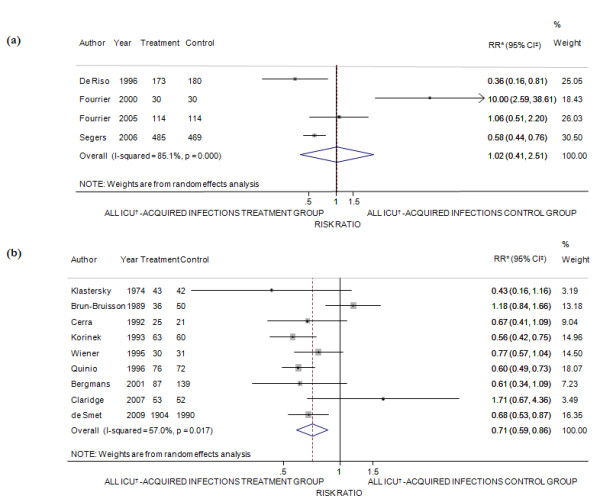
**Meta-analysis of the effectiveness of topical SDRD in the prevention of all ICU-acquired infections**. (**a**) Decontamination by antiseptics. (**b**) Decontamination by antibiotics. * Risk ratio; ^‡ ^confidence interval; ^† ^intensive care units.

Four studies on antiseptics contributed to the analysis of all ICU-acquired infections prevention [51,52,54,56] (Figure 3) and indicated no statistically significant beneficial effect of the experimental treatment (efficacy = -2%; 95% CI of efficacy = -151% to 59%; Q = 20.14 *P *< 0.001; I^2 ^= 85.1%). If the analysis was restricted to high quality studies, the efficacy to prevent all ICU-acquired infections was 41% (95% CI of efficacy = 24% to 53%) and a reduction of heterogeneity among studies became evident (Q = 3.9 *P *= 0.14; I^2 ^= 48.7%). Similarly, studies involving only specialty surgery ICU showed a significant decrease of all ICU-acquired infections, with a value of 45% (95% CI of efficacy = 28% to 57%).

Nine trials that tested antibiotic prophylaxis were available for the analysis of all ICU-acquired infections [37,38,58,60,64-68] (Figure 3) and a significant decline in all ICU-acquired infection rate was demonstrated (efficacy = 29%; 95% CI of efficacy = 14% to 41%; Q = 18.62 *P *= 0.02; I^2 ^= 57%). Sensitivity analyses proved an efficacy of the treatment only in the high quality studies when only the digestive tract decontamination was taken into account, when the analysis was restricted to mixed ICU, and to similar conclusion led the meta-analyses limited to research that used the same antibiotic combinations, cyclic peptide plus aminoglycoside plus polyene antifungal drug and vancomycin plus other antimicrobial agents. In most restricted analyses, heterogeneity disappeared.

#### Other VAP related outcomes

VAP is important not only if it increases mortality, but also the length of hospital stay, duration of mechanical ventilation, use of antibiotics, quality of life and the length of ICU stay. We tentatively tried to extract data on some of these outcomes from the included trials and we could perform meta-analyses on the duration of mechanical ventilation and length of ICU stay. However, data on duration of mechanical ventilation were available only in two antiseptics trials [36,55] and in four antibiotics trials [62,67,69,70] and gave a non-significant effect with a mean difference (MD) for antiseptic use of -0.19 days (95% CI = -0.46 to 0.07; Q = 0.07 *P *= 0.79; I^2 ^= 0%) and for antibiotic use of -0.12 days (95% CI = -0.34 to 0.11; Q = 0.54 *P *= 0.91; I^2 ^= 0%). Data on length of ICU stay were available in three studies using antiseptics [36,55,56] and in six studies using antibiotics [60,62-64,67,69]. Pooled analyses of these trials did not show an influence on the mean difference of length of ICU stay both for antiseptics (MD = -0.7 days; 95% CI = -0.19 to 0.04; Q = 0.4 *P *= 0.81; I^2 ^= 0%) and antibiotics (MD = -0.34 days; 95% CI = -0.73 to 0.05; Q = 27.67 *P *< 0.001; I^2 ^= 81.9%).

#### Publication bias

Funnel plots showed no significant asymmetry for studies exploring the preventive role of antiseptics and of antibiotics.

## Discussion

### VAP

The major finding of the present meta-analysis provides support to the observation that topical antiseptic or antibiotic SDRD plays a significant role as a protective factor against the development of VAP.

Chlorhexidine has been used as a degerming agent in all but one antiseptic trial, because it has a high level of antibacterial, antiviral and antifungal activity, it is virtually free of adverse effects [72] and it is an inexpensive solution. Seguin *et al*. used povidone-iodine, another antiseptic agent that has high, rapid and persistent activity on Gram-negative and Gram-positive bacteria [73,74], and it is simple and safe to use [75]. Our results do not allow us to determine which of the two antiseptics has a greater effect, but confirmed the preventive role of antiseptics against VAP. To find the best disinfectant further studies are needed comparing povidone-iodine and chlorhexidine in preventing VAP in "head to head" trials.

The findings of this pooled analysis are consistent with the relatively strong protective association observed between antiseptic oropharyngeal decontamination and the risk of VAP observed in previous meta-analyses [25-28].

Since the pathogenesis of VAP is related to contamination of the aero-digestive tract, our meta-analyses on antibiotics were conducted combining data on topical digestive and respiratory tract decontamination and our findings showed a significant protective effect of the antibiotics. This result persisted when we considered separately the digestive and the respiratory tract decontamination, suggesting that any of these two ways of decontamination may be used. Only two studies [57,70] classified VAP according to time of onset in early and late-onset [76]; therefore, we have not performed a sensitivity analysis on this issue.

Comparisons with the results of previous meta-analyses are very difficult since the inclusion criteria used were different. Indeed, Liberati *et al*. [31] did not make any restriction on the type of respiratory tract infections (RTIs), combining trials on pneumonia and tracheobronchitis in ICU patients, nor on unpublished RCT or on language, whereas they restricted inclusion to digestive decontamination by antibiotics only. The results of Liberati *et al*. showed a significant protective effect on RTIs.

Chan *et al*. [27] did not reach our conclusions since their results suggested no significant protective effect of topical antibiotic prophylaxis on VAP. Indeed, the meta-analysis of Chan *et al*. had only two studies [68,71] in common with ours, and pooled 1,098 patients, whereas the present meta-analysis could take into account a larger sample size (2,463 patients). Finally, Falagas *et al*. [30], who took into account only the effect of administration of antimicrobial agents via the respiratory tract, concluded that pneumonia occurred significantly less often in the prophylaxis arm compared to the comparison arm. Our sub-analysis on trials that tested respiratory tract decontamination confirms these results, even if the sample analyzed is different because we included only pneumonia occurring in patients assisted by mechanical ventilation; therefore, we excluded the trials of Greenfield *et al*. [77] because data on colonization only was reported and of Klick *et al*. [78] for incomplete data on outcomes. Moreover, we excluded non-randomized trials [79] and abstracts [80], and included one study in which the preventive strategy was topical decontamination in the subglottic area [70], and a recent trial that tested prophylactic administration of aerosolized ceftazidime [37].

### Mortality

In the present meta-analysis neither antibiotic nor antiseptic topical decontamination influenced overall mortality, and the results did not substantially differ in the separate meta-analyses performed according to different quality and study design or details of intervention. Liberati *et al.*, in previously published meta-analysis, examining the prophylactic use of antibiotics, have shown efficacy in reducing mortality when topical decontamination was combined with intravenous antibiotic administration, whereas, in line with our results, topical prophylaxis alone was not effective. No former meta-analyses on antiseptics or antibiotics [27-29,31] showed a significant beneficial effect on mortality.

These results could be due to lack of an effect of administration of topical agents on mortality since in only one trial that used antiseptic decontamination [51], and in no antibiotic trials [37,38,57-71], was a significant reduction in mortality found. Another possible explanation was that in most of the trials mortality was a secondary outcome and data accuracy could be lower than primary outcome; therefore, analysis combining these data could have failed to show an effect of experimental treatment. Also, in our case the overall sample size was small and, therefore, could have limited the interpretation of the effect on mortality. Another possible explanation can be related to our inability to distinguish the role of the topical SDRD on the occurrence of early and late-onset VAP. It has been demonstrated that VAP related mortality is restricted mainly to patients with late-onset VAP; it is only marginally reduced with appropriate empirical antibiotic treatment [14]. In the same study the authors report that the prophylactic regimen they used, that is, the universal use of continuous aspiration of subglottic secretions, was recognized to be a reducing and delaying factor for VAP. Therefore, we may hypotheses that if the topical SDRD is also particularly effective only on the reduction of early-onset VAP, its role on late-onset VAP related mortality may be marginal. Trials allowing data extraction on the occurrence of early and late-onset VAP separately are strongly needed.

### All ICU-acquired infections

To the best of our knowledge, this is the first meta-analysis aimed at the assessment of the effectiveness of topical SDRD on the incidence of all ICU-acquired infections.

Our results have shown the effectiveness of topical SDRD both with antiseptics, although only in the sensitivity analyses restricted to high quality studies and to those involving only specialty surgery ICU, and antibiotics.

These achievements confirmed what we expected because colonization is a prerequisite for the development of infections that frequently arises from the endogenous flora in the oropharyngeal and intestinal tract; therefore, healthcare-associated infections were potentially preventable through the suppression of colonization of the digestive and/or the respiratory tract. Also, nosocomial pneumonia is the second most common healthcare-associated infections (HAI) and the most frequently acquired infection in the ICU, so all ICU-acquired infections could be reduced secondarily from a VAP prevention.

### Resistance to antiseptic and antimicrobial agents

Considering the importance of antibiotic-resistance in ICUs, we focused our interest on topical administration since it has been reported that it is more frequent when a combination of topical plus systemic antibiotics was used [81,82] rather than only topical antibiotics [38,83]. Anyway, the biggest criticism even against topical SDRD is the emergence of resistant strains. This issue has been only marginally investigated in most of the studies included in the meta-analysis. The majority of the trials using antibiotics [37,38,57,59,61,62,64,68,69] found no increase in infections caused by antibiotic-resistant organisms, at least during the relatively short period of studies, whereas a trend towards increased colonization of patients by resistant microbial strains was reported [58,66,67]. This increase was not found in studies using antiseptics [37,51,52,57]. On the basis of these results, a long-term increase in the occurrence of HAIs sustained by resistant strains as a result of topical SDRD with antibiotics cannot be excluded, and this issue warrants cautious attention in further studies. However, it should be pointed out that routine five-year use of selective digestive decontamination was not associated with increased antimicrobial resistance rates [83]. This finding allows us to suggest an analogous result with the use of long-term topical SDRD at least in ICU with low baseline resistance rates.

### Strengths and limitations of the study

The strengths of the present meta-analysis include the considerable number of studies and subjects included as well as the acceptable methodologic quality of the studies on which the analysis is based. It is well-known that the quality assessment of the primary studies has been identified as one of the most important steps of the peer-review process [84]. This comes from the consideration that studies of poor quality may yield information that is not valid; therefore, the inclusion of studies with invalid information in a meta-analysis can make the conclusion of the meta-analysis invalid. Therefore, taking the quality of studies into account in a meta-analysis has the potential to enhance the validity of a meta-analysis because quality is implicitly a measure of validity [85]. Moreover, the evaluation of the quality of the studies in a meta-analysis may contribute to point out limitations in published studies and suggest ways to improve the methodology of studies in further research. In this meta-analysis the quality of the RCTs was good with regard to the various methodological aspects of the research protocol (for example, description of therapeutic regimen, criteria for patient selection, randomization, blinding) while the main shortcomings were related to the overall assessment of the statistical methods and presentation of data. Limitations of the present meta-analysis study include the heterogeneity between the studies with respect to patient populations that had a different profile of risk factors; different medications used, particularly in antibiotic trials, in the choice of the antimicrobial agents or their associations or the delivery mode; different approaches for the control arms and different outcome definitions. Moreover, among our inclusion criteria there was restriction to studies published in English. This is a controversial issue, since some argue that authors are more likely to report positive results in international journals and negative results in local journals, as demonstrated by Egger *et al*. [86]. Language restriction could, therefore, introduce bias in the results of the meta-analysis [87,88]. However, there has also been evidence that studies published in local journals may be of lower methodological quality, as reported by Sterne *et al*. [89], and this would be in favor of their exclusion. We have also performed a search without language restriction and it would have led to the inclusion of two more papers [90,91]. Vogel *et al*. [90] was excluded because it was not a RCT, while we have repeated the meta-analyses of topical SDRD by antibiotics on VAP prevention and on mortality by entering Rathgeber *et al*. [91] and the results did not alter our conclusions. Indeed, the overall estimate of efficacy of antibiotics on VAP prevention was 37% (95% CI of efficacy = 20% to 51%) and the meta-analysis on mortality showed no significant effect (efficacy = -2%; 95% CI of efficacy = -12% to 7%). These results were confirmed in the sensitivity analyses involving only upper respiratory tract decontamination with antibiotics (data not shown). Therefore, we believe that our strategy is not prone to substantial bias or to low robustness in the overall results. Finally, the findings are affected by the limitations of the individual trials included. According to the mentioned limitations, the results of the meta-analysis must be interpreted with caution; however, the careful examination of possible sources of heterogeneity contributed to assess the methodologic quality of research, and to identify potential biases, data gaps, and suggestions for future research.

## Conclusions

In conclusion, despite the above limitations, we think that our results prove that topical SDRD using antiseptics or antimicrobial agents is effective in reducing the frequency of VAP in ICU. Unlike antiseptics, the use of topical antibiotics seems to be effective also in preventing all ICU-acquired infections, while the effectiveness on mortality of these two approaches needs to be investigated in further research. Also, further research is essential to compare different preventive protocols in ICU patients, such as the "head to head" comparison of topical antiseptics and antibiotics, the oral cavity decontamination only compared to the whole digestive tract, or the decontamination of the airways to the digestive tract. Finally, a more careful assessment of the cost-effectiveness of preventive interventions used and a more systematic evaluation of issues related to the emergence of drug resistance are necessary.

## Key messages

■ VAP is related to a high rate of morbidity, complications, prolonged ICU stay and mortality in patients receiving mechanical ventilation.

■ Colonization of the aerodigestive tract is primarily involved in VAP's pathogenesis and represents a main objective for prevention.

■ Topical SDRD using antiseptics or antibiotics is effective in reducing the incidence of VAP in ICU.

■ Topical SDRD using antibiotics is effective in reducing the incidence of all ICU-acquired infections.

■ Further research is essential to compare different preventive protocols in ICU patients and to assess the cost-effectiveness of preventive intervention used.

## Abbreviations

CI: confidence intervals; HAIs: healthcare-associated infections; MD: mean difference; OR: odds ratio; RCTs: randomized controlled trials; RR: risk ratio; RTIs: respiratory tract infections; SDRD: selective digestive or respiratory tract decontamination; VAP: ventilator-associated pneumonia.

## Competing interests

The authors declare that they have no competing interests.

## Authors' contributions

CP participated in the conception and design of the study, collected the data, contributed to the data analysis and its interpretation, and wrote the first draft of the article. AB, DF and CGAN collected the data, and contributed to the data analysis and interpretation. MP designed the study, was responsible for the data analysis and interpretation, and wrote the article. CP and MP are guarantors for the study. All authors had full access to all of the data (including statistical reports and tables) in the study and take responsibility for the integrity of the data and the accuracy of the data analysis. All authors have read and approved the manuscript for publication.
